# A Computational Model of Cellular Engraftment on Lung Scaffolds

**DOI:** 10.1089/biores.2016.0031

**Published:** 2016-10-01

**Authors:** Joshua J. Pothen, Vignesh Rajendran, Darcy Wagner, Daniel J. Weiss, Bradford J. Smith, Baoshun Ma, Jason H.T. Bates

**Affiliations:** ^1^ University of Vermont College of Medicine, Burlington, Vermont.; ^2^ Comprehensive Pneumology Center, Ludwig-Maximilians-Universität, Universitätsklinikum Grosshadern, und Helmholtz Zentrum München, München, Germany.

**Keywords:** agent-based model, image processing, lung scaffold, stem cells

## Abstract

The possibility that stem cells might be used to regenerate tissue is now being investigated for a variety of organs, but these investigations are still essentially exploratory and have few predictive tools available to guide experimentation. We propose, in this study, that the field of lung tissue regeneration might be better served by predictive tools that treat stem cells as agents that obey certain rules of behavior governed by both their phenotype and their environment. Sufficient knowledge of these rules of behavior would then, in principle, allow lung tissue development to be simulated computationally. Toward this end, we developed a simple agent-based computational model to simulate geographic patterns of cells seeded onto a lung scaffold. Comparison of the simulated patterns to those observed experimentally supports the hypothesis that mesenchymal stem cells proliferate preferentially toward the scaffold boundary, whereas alveolar epithelial cells do not. This demonstrates that a computational model of this type has the potential to assist in the discovery of rules of cellular behavior.

## Introduction

One of the hopes of stem cell research is being able to grow entire organs *ex vivo* for use in clinical transplantation and as model systems for study. An approach that may hold promise for the engineering of complex organs is the use of decellularized scaffolds devoid of cells and antigenic material that are then seeded with autologous stem cells in the hope that a fully functional organ will eventually develop.^1,2^ In the case of the lung, as with many organs, this promise remains far from realized.^3,4^ Indeed, stem cell research has thus far been essentially empirical, leading some to refer to these investigations as “modern alchemy.”^1^ One possible reason for the current state of affairs is an almost complete lack of predictive modeling tools with which to guide the direction of investigation.

The ability to predict the course of tissue regeneration must rely on a knowledge of the rules governing how relevant cell types behave in the various situations they will encounter during the regeneration process. These rules indicate how each cell influences, and is influenced by, its neighboring cells and the surrounding microenvironment in which it finds itself.^5,6^ Armed with such rules, one could, in principle, simulate the process of tissue regeneration to identify promising scenarios before trying them experimentally, thereby greatly increasing the efficiency of the search for effective regeneration strategies. The computational approach known as agent-based modeling^7–10^ seems perfectly suited to this endeavor. However, such modeling relies on knowledge of the rules of cell behavior, about which we currently know little. On the other hand, agent-based modeling might also be of assistance in determining these rules through comparison of model predictions to experimental data.

Sophisticated agent-based models have been applied to a variety of cellular systems, including the growth and maintenance of skin^11^ and the use of mesenchymal stem cells (MSCs) to generate bone.^12^ In the case of bone, models have been applied specifically to the problem of understanding how cells attach to and migrate through porous scaffolds.^13^ However, computational models have yet to be developed for the study of stem cell behavior in scaffolds created by decellularizing lung tissue. Accordingly, we investigated the engraftment patterns of two different cell types relevant to the regeneration of lung tissue following their seeding onto decellularized lung scaffolds. Simultaneously, we employed an agent-based computational model to simulate engraftment patterns based on simple sets of behavioral rules governing how cells move, when they divide, and when they die. By determining which rules more closely recapitulated experimental observations, we are able to infer those that plausibly might differentially regulate the behavior of the two cell types.

## Methods

### Computational model

We created a computational model to study two computational Hypotheses, termed Hypotheses 1 and 2, respectively, regarding the behavior of stem cells on these decellularized scaffolds. Both hypotheses are motivated by experimental observations regarding two representative cell types investigated for seeding decellularized lung scaffolds: C10 epithelial cells, an immortalized mouse type 2 alveolar epithelial cell line, and bone marrow-derived MSCs.^14,15^ From previous experiments, we know that C10 cells tend to mostly be present at the tissue periphery at later time points.^14^ Given the nature of cells to move through chemotaxis, this suggests that there is some substrate incorporated into the scaffold with a concentration that is highest at the borders of the tissue slice and decreases progressively toward the center. The identity of this substrate is currently unknown, but possibilities include oxygen or extracellular matrix components such as fibronectin and laminin, which may be distributed preferentially toward the periphery of the alveolar tissue.^16^

We used NetLogo 4.1.3 freeware^9,10^ to design a three-dimensional (3D) agent-based model of a decellularized scaffold environment seeded with cells that can attach to and proliferate over it. The environment of the model represents an initially decellularized lung scaffold and is composed of a set of contiguous cuboidal patches, each characterized by local variables that define its properties. The cells applied to the model scaffold are represented by discrete agents capable of moving around from patch to patch according to stochastic rules that define the likelihood of their rates and directions of movement.

**FIG. 1.**
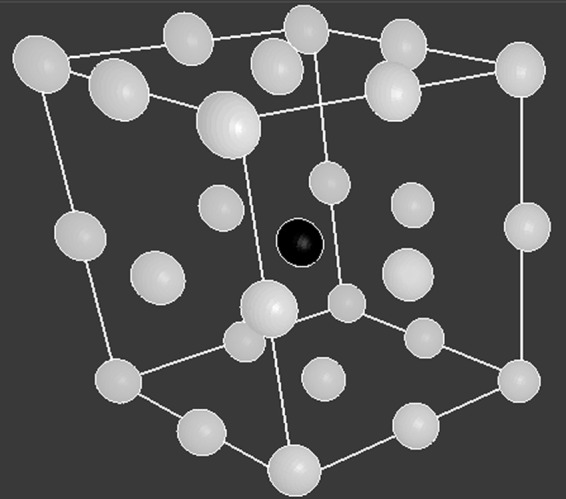
Diagram illustrating the location of a cell's neighbors. The cell (black ball) is at the center of the cube, and the 26 white balls illustrate the location of its neighbors in 3D space. Each cell has up to 26 neighbors. 3D, three dimensional.

At every time point, only one cell can exist on each patch, meaning a motile cell cannot move into the same location as an already engrafted cell. However, additional rules allow the cells to interact with neighboring agents and the patches that they come into contact with. Each cell has up to 26 neighbors as illustrated in Figure 1. These interactions may involve any putative biological effect such as the alteration of the environment by the secretion of chemical signals or a direct effect of one cell on another. In particular, we assume that engrafted cells increase the substrate concentration of the patch they are on by an arbitrary value of 1. If the cell dies, then the patch substrate concentration is decreased by 1.

We represent the environment of the lung scaffold as 32 × 32 × 32 individual cubic patches, with the environmental border representing the edges of the scaffold. (The environment size and the spacing between the patches are constraints set by the NetLogo software.) Each patch has a local variable whose value represents the numerical concentration of a bioactive substrate that influences cell behavior (see Rule 1 below) such that the substrate concentration has a value c = ${ \frac { 20 \sqrt { x^2 + y^2 } }  { 21.2 } }$ for any set of (*x*, *y*, *z*) coordinates, as the *x*, *y*, and *z* axes range from −16 to 16 (in arbitrary distance units). While this formula is arbitrary, it creates an environment where in any *x*-*y* plane, the lowest substrate concentration (c = 0) is at the center (*x* = 0, *y* = 0), while the highest concentration is at the borders, thereby creating a substrate gradient and thus an impetus for cell movement and exploration of the environment.

The scaffold was seeded at *t* = 0 with 30,000 randomly placed identical agents (cells) because this gave a seeding density that was visually reminiscent of the experimental situation. Experimentally, we observe that most cells seem to engraft following initial seeding, at least for a while, so the cells in the model were each given a 95% chance of engrafting with the remaining cells being eliminated. The model was then run for 80 time steps using two different behavioral rules sets corresponding to computational Hypotheses 1 and 2. Each cell executes its set of commands once within each time step, allowing it to potentially move to a neighboring patch, engraft to a patch, and/or undergo one round of proliferation. We ran the model for a total of 80 time steps because most of the cells in the model under both hypotheses had died by this time point, which matches what we observed experimentally (see Fig. 6).

**FIG. 2.**
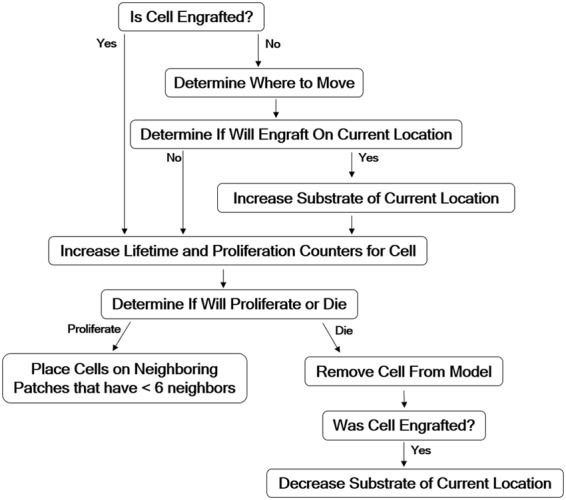
Schematic of the algorithm used with specific rule sets at each time point to implement Hypotheses 1 and 2 in the model.

We show a schematic for the general behavior of the models in Figure 2. The specifics of the model using the rule set for Hypothesis 1 are as follows:

(1) First, the motile cells determine where to move across all three axes. While each cell has 26 neighbors surrounding it (Fig. 1) and there are numerous ways of modeling how it could move and the number of neighboring patches it could consider moving to, cell movement in NetLogo is modeled as a forward vector oriented randomly in some direction relative to the *x*, *y*, and *z* axes, thus enabling the cells to explore their 3D environment by moving and considering neighbors in a two-dimensional (2D) manner along the direction of the forward vector. The angle of the vector is randomly changed at each time step, changing the cell's orientation to the *x*, *y*, and *z* axes, unless the cell senses a high substrate signal in one of the three patches ahead of its current direction (directly ahead, ahead to the left, and ahead to the right), in which case the cell has a higher chance of moving toward the patch with the strongest signal. (Because of the angle orientation, these three patches may be above or below the cell's current location.) To simulate this in our model, the numerical variables representing concentrations of substrate, *c*, on these three patches are each multiplied by a random number, *x_i_*, uniformly distributed on the interval [0, 1], which represents the likelihoods of moving in each of the directions. If *S* = (*x*_1_*c_ahead – and – straight_* + *x*_2_*c_ahead – and – left_* + *x*_3_*c_ahead – and – right_*) > 1, the cell moves toward the patch with the strongest signal, meaning that all cell movements in the model are probabilistic. If *S* < 1, the cell moves randomly to an adjacent patch. (The concentration of substrate on the current patch is *c_patch_*.) The rules governing cell movement are based on previous models regarding cellular movement throughout the lung in the presence and absence of chemical stimuli.^9,10^
(2) All motile cells then determine whether they will attach to the patch they are currently on. A random real number, *N*, uniformly distributed on the arbitrary interval [0, 100] is computed. If *N* > *N_crit_*, where *N_crit_* = 5, the cell is replaced by an immobile engrafted cell. Each engrafted cell has two parameters: *T_life_*, the lifespan of the cell, and *T_prol_*, the amount of time that must pass before the cell can proliferate into neighboring patches. Upon initially engrafting, *T_life_* is chosen randomly on the uniformly distributed interval of [0, 36] time steps, and *T_prol_* is set on the uniform interval [33, 87]. (This enables some cells to undergo apoptosis before having the opportunity to proliferate, and to have an increased likelihood of proliferating if the patch environment enables them to proliferate in a shorter amount of time.)
(3) The lifetime counter, *N_life_*, for the cell is incremented by 1. If *N_life_* = *T_life_*, the cell is eliminated from the model, representing cellular apoptosis.
(4) The proliferation time counter, *N_prol_*, for the cell is incremented by 1. If *N_prol_* = *T_prol_*, the cell places a copy of itself onto a single randomly selected neighbor that has at most six neighbors itself. (The number of neighbors is arbitrarily chosen, but is based on observations from our experimental images that cells that survive over time are surrounded by a small number of cellular neighbors. This suggests that cells require some moderate degree of cell–cell interaction to thrive.) For each new cell, *T_life_* is chosen randomly on the interval [0, 18] time steps, and *T_prol_* is set to (33 − *c_patch_*/*c_envfactor_*) + *n*, where *c_envfactor_* = 1.9 and *n* is a random number uniformly distributed on the interval [0, 54].
(5) As long as an engrafted cell remains alive, *c_patch_* is increased by *c_amount_*. This implies that the engrafted cell through its actions, either directly or indirectly, permanently increases the concentration of substrate on the patch by *c_amount_*. If the cell dies, then it is no longer able to maintain the substrate concentration at this level; we assume that it decays somewhat, so *c_patch_* is decreased by *c_amount_*. In our model, *c_amount_* is set to 1.

Under the rule set for Hypothesis 1, engrafted cells have an equal probability of surviving anywhere in the environment, but a higher likelihood of proliferating on patches with higher concentrations of substrate. The rule set for Hypothesis 2 is identical to Hypothesis 1, with the exceptions that *T_life_* is distributed uniformly on the interval [0, 45] and *T_prol_* is set on the uniform interval [41, 103], and for each new cell created, *T_life_* is set to (*c_patch_*/*c_envfactor_*) + *n*, where *c_envfactor_* = 1.9, *n* is a random number uniformly distributed on the interval [0, 45], and *T_prol_* is chosen randomly on the uniformly distributed interval [41, 103] time steps. Under this hypothesis, cells live longer relative to the cells in Hypothesis 1, and engrafted cells have a higher likelihood of surviving on patches with higher concentrations of substrate.

### Experimental data

Cells were seeded onto lung scaffolds from mice as described in Bonenfant et al.^14^ and Wallis et al.^15^ Briefly, the study utilized adult C57BL/6J mice that were maintained at UVM in accordance with institutional and American Association for Accreditation of Laboratory Animal Care standards and review. Following heart–lung bloc harvest, lungs were decellularized under sterile conditions by tracheal and vascular infusion, and immersion in 0.1% Triton-X solution (Sigma-Aldrich, St. Louis, MO) for 24 h and 2% sodium deoxycholate (Sigma-Aldrich) for a further 24 h. Between each incubation step, lungs were rinsed with 5× penicillin/streptomycin (P/S; Cellgro) in deionized water. On day 3, lungs were incubated for 1 h each in 1 M NaCl and porcine pancreatic DNase solution (Sigma-Aldrich).

Lungs were then rinsed with 1× phosphate-buffered saline in 1× P/S. The left lobe of decellularized scaffolds was then inoculated through the airway with either 1× 10^6^ MSCs isolated from mouse bone marrow (Sca-1^+^, CD106^+^, CD29^+^ and CD11b^−^, CD11c^−^, CD34^−^, and CD45^−^, from Dr. Darwin Prockop, NCRR/NIH Center for Preparation and Distribution of Adult Stem Cells at Texas A and M University),^17^ or 1 × 10^6^ C10 mouse lung epithelial cells (gift from Dr. Matthew Poynter, University of Vermont). Cells were suspended in 3% low melting point agarose (SeaPrep Agarose; Cambrex, East Rutherford, NJ) for inoculation. The inoculated lungs were then allowed to gel at +4°C for 30 min and manually sliced to sections of ∼1 mm thickness.

Slices were then incubated at 37°C in respective growth media to remove the agarose. Murine bone marrow-derived mesenchymal stromal cells (mMSCs) (P6-P8) and slices inoculated with mMSCs were cultured in Iscove's Modified Dulbecco's Medium (Sigma-Aldrich) supplemented with 2 mM l-glutamine, 100 U/mL penicillin and 100 mg/mL streptomycin (Fisher Scientific, Waltham, MA), 10% fetal bovine serum (FBS; Atlanta Biologicals, Flowery Branch, GA), and 10% horse serum (Invitrogen, Carlsbad, CA). C10 cells and slices inoculated with C10 cells were cultured in Dulbecco's modified Eagle's medium (Sigma-Aldrich) supplemented with 2 mM glutamine (Invitrogen), P/S, and 10% FBS.

Media were changed every other day and individual slices were incubated at 37°C at 5% CO_2_ in low adherence 12-well tissue culture plates. Scaffolds were harvested at days 1, 3, 7, 14, 21, and 28 after inoculation and fixed in 4% paraformaldehyde (Sigma-Aldrich). Slices were then embedded in paraffin and sliced to 5-μm-thick slices, deparaffinized, and stained with hematoxylin and eosin (Electron Microscopy Sciences, Hatfield, PA).

### Image analysis

Images were taken from the top, middle, and bottom of each experimental lung slice using an Olympus fluorescent microscope at 10× magnification. At each time point, we imaged three slices, for a total of nine experimental images per time point. In our computational analysis, we took 30 screenshots of 2D slices from the 3D computational model scaffold every 10 time steps.

We defined our model as having a total time duration evenly divided into 80 time steps. The time scale in this model is arbitrarily defined, but we can nevertheless map the speed of movement of a model cell from patch to patch onto the actual speed of movement of real cells, as follows: a model tissue slice consists of 32 × 32 patches corresponding to an area of lung scaffold roughly 1,000 × 1,000 μm; so each model patch has dimensions roughly 30 × 30 μm. As 80 time steps in the model correspond to 28 days of real time, a cell moving from patch to patch at each time step has a velocity of 0.002 time step/min, corresponding to an actual velocity of about 0.06 μm/min. This is of the same order of magnitude as we estimate the velocity of movement of cells engrafted to lung scaffolds (unpublished observations).

**FIG. 3.**
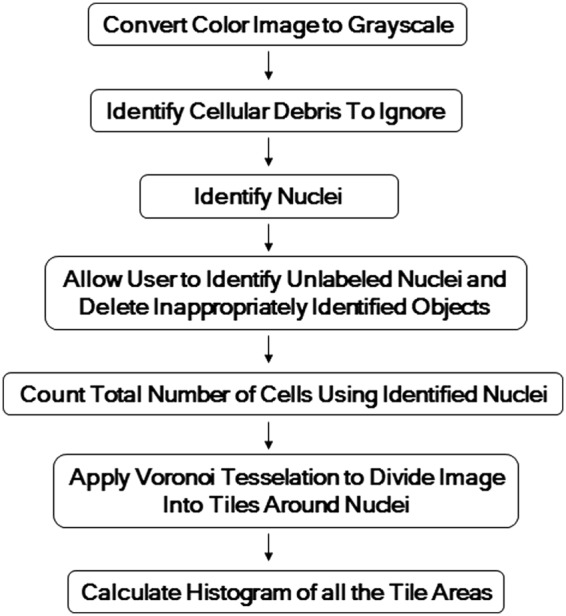
Illustration of the image analysis procedure used to define cell neighborhoods using Voronoi tesselation.

We quantified the patterns of cellular adherence in the images from both the experimental lung scaffolds, and the agent-based model using a custom-designed image analysis tool written in Matlab (Mathworks, Natick, MA) identifies cell nuclei and divides the image into tiles centered on them by implementing the following algorithm. (The Matlab code is available upon request from the authors.) A schematic of this algorithm is shown in Figure 3, and the details are as follows:

(1) Convert the color image to grayscale.
(2) Create a black and white version of the grayscale image using a pixel intensity threshold of 125 on an 8-bit scale from 0 (black) to 255 (white). Identify contiguous components of at least 300 pixels (considered to be a mixture of cellular clumps and debris) from the black and white image, which become the image's “mask” regions.
(3) Convert the original grayscale image to a black and white image using a pixel intensity threshold value of 125. Outside the “mask” regions determined in Step 2, mark the centroids of all connected components of at most 20 pixels (likely to be cell nuclei).
(4) Repeat Step 3, but with a pixel intensity threshold value of 85, and instead mark centroids within the “mask” regions.
(5) Allow user to manually edit cell identification by including missed cells and deleting inappropriately included objects.
(6) Count the total number of cells in the image by counting the number of cell nuclei.
(7) Apply Voronoi tessellation^18,19^ such that each cell nucleus lies at the centroid of a polygon-shaped tile that defines the local neighborhood of that cell.
(8) Calculate variance of the histogram of Voronoi tile areas.

**FIG. 4.**
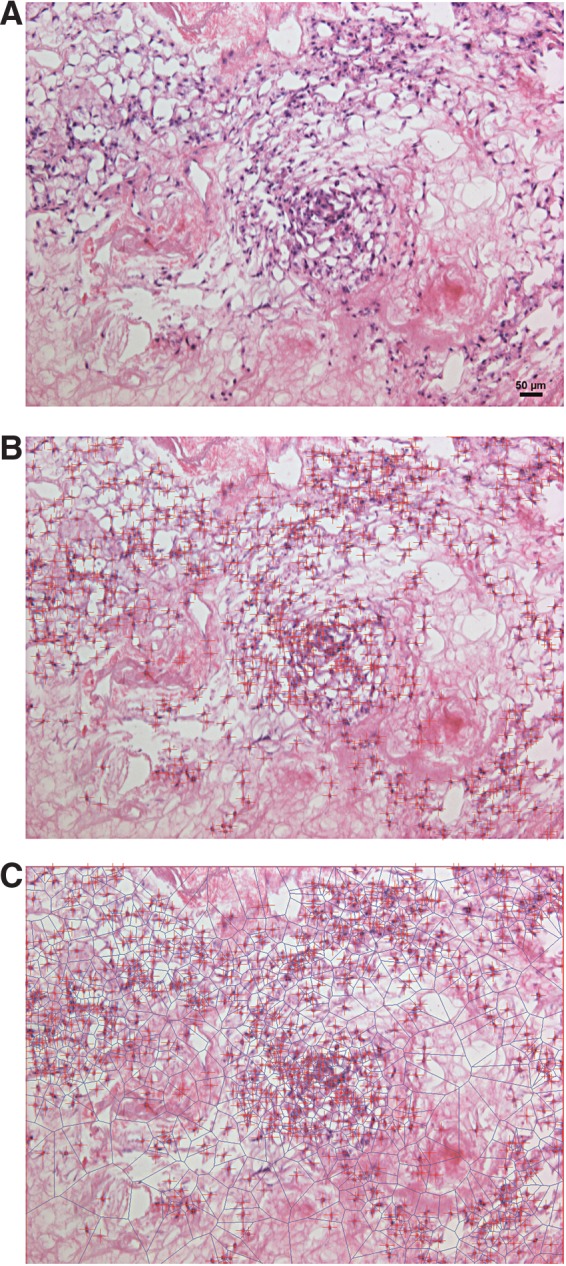
Experimental image **(A)**, which is processed to mark all cells with crosses for counting **(B)** and then undergoes Voronoi tessellation to split the region into tiles centered around each cell **(C)**.

Figure 4 shows an example of the image processing algorithm applied to an image of a slice from one of these experimental lung scaffolds, illustrating how the Voronoi tessellation defines the neighborhood of each cell. In particular, note that the areas of the tiles around isolated cells are larger than those around cells that are clustered.

### Statistical analysis

The results of the cell culture experiments (C10 cells vs. MSCs) and the computational modeling (Hypothesis 1 vs. Hypothesis 2) were compared by two-way analysis of variance (ANOVA). Statistical significance was taken as *p* < 0.05. The sensitivity of model predictions to variations in model parameter values was assessed by one-way ANOVA.

## Results

**FIG. 5.**
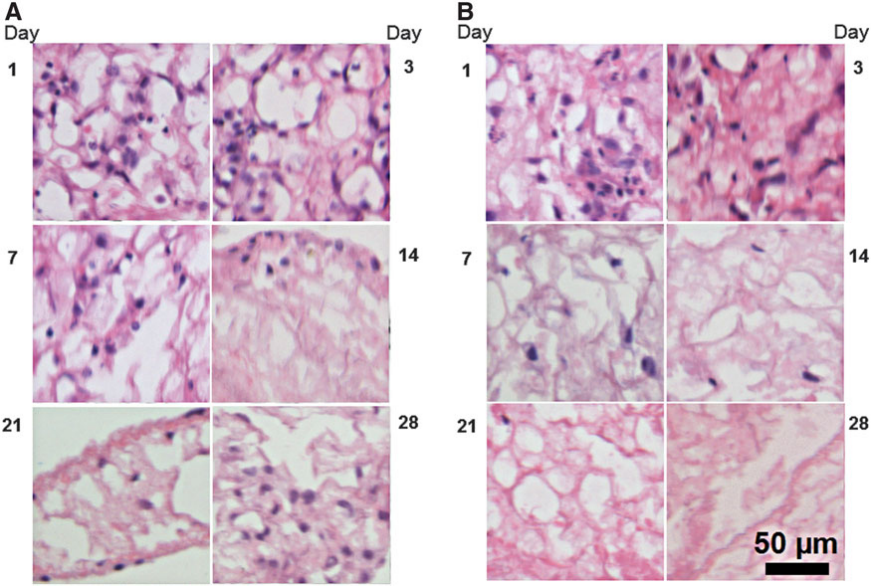
Experimental images of decellularized lung scaffolds on days 1, 3, 7, 14, 21, and 28, following seeding with **(A)** C10 epithelial cells and **(B)** MSCs. MSCs, mesenchymal stem cells.

**FIG. 6.**
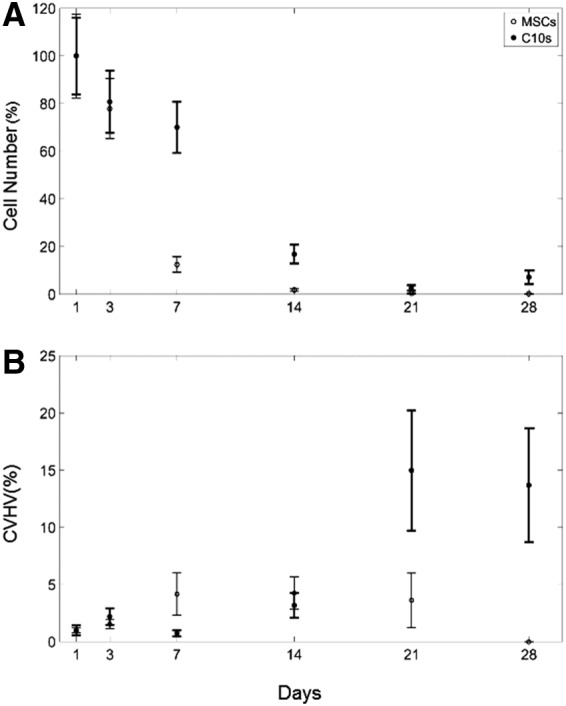
Experimental results (mean ± SE) for **(A)** number of cells, and **(B)** CVHV for cells following the rules for MSCs (open circles) and C10 epithelial cells (closed circles) for all nine images gathered on days 1, 3, 7, 14, 21, and 28. CVHV, Corrected Voronoi Histogram Variance; SE, standard error.

Sample images of the decellularized scaffolds at various time points following seeding with C10 cells or MSCs show evolving patterns of cellular engraftment and proliferation across these scaffolds (Fig. 5A, B, respectively). We applied our image analysis tool to the 54 images (at 6 time points taken from 9 separate scaffold images) acquired over the 28-day time course with the two experimental cell types. Figure 6A shows the number of cells (mean ± standard error [SE]) from the nine scaffold images versus time after inoculation, where the results from each scaffold have been normalized to their respective values on Day 1. Both C10 cell and MSC numbers eventually approach zero, but at different rates and were statistically significantly different (*p* < 0.001). Specifically, the MSCs have fallen off markedly by about day 7, whereas the C10 cells take about twice as long to reach correspondingly low numbers.

It should be noted that both cell types eventually die out completely, meaning that attempts to engraft the scaffold with persistent cells (i.e., cells that survive and remain engrafted throughout the time course of the experiment) were ultimately unsuccessful. This speaks of the inherent difficulties of tissue regeneration in general and the fact that in the case of the lung, this field is still in its infancy.

Cell numbers and Corrected Voronoi Histogram Variance (CVHV) are expressed as percentages of their respective values at the initial time point. At the first time point, the MSC cell number was 343.33 ± 60.27 and CVHV was 2.93 ± 5.26 × 10^5^, while the C10 cell number was 692.44 ± 111.82 and CVHV was 2.07 ± 8.84 × 10^9^.

We also determined the variance of the histogram of the Voronoi tile areas at each time point. This variance gives a measure of the heterogeneity of cell clustering, but images with greater numbers of cells inherently have smaller variance values compared to images with fewer cells because more cells means a smaller mean area per cell. To control for this, we multiplied the histogram variance by the number of cells in the image to derive what we term the CVHV. Figure 6B shows that the CVHV (mean ± SE, with the results from each scaffold normalized to their respective values on Day 1) versus time for the two cell types begins to diverge at about day 14, and is markedly different by the end of the experiment. The CVHV values for the two cell types were statistically significantly different (*p* = 0.0064).

**FIG. 7.**
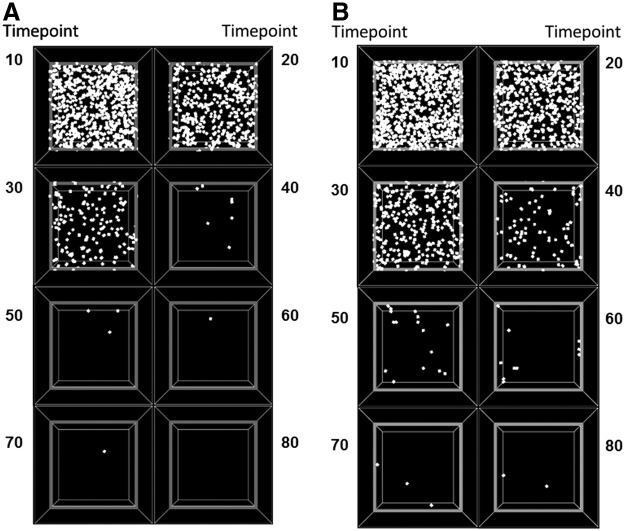
Screenshots of a computational model of a 3D lung scaffold at 10, 20, 30, 40, 50, 60, 70, and 80 time steps, following seeding with cells that behave according to **(A)** Hypothesis 1 (shorter lived cells with preferential proliferation on areas of higher substrate) and **(B)** Hypothesis 2 (longer lived cells with preferential survival on areas of higher substrate).

We performed a corresponding procedure on images generated by the agent-based model, where 80 time steps in the model correspond roughly to 30 days of experimental time as judged by the relative numbers of surviving cells at these respective time points. Patterns of cellular proliferation and spreading throughout a simulated 2D slice of scaffold created by the agent-based model, using the cellular rules specified by Hypotheses 1 and 2, are shown in Figure 7A and B, respectively. Similar numbers of cells initially engraft to the scaffold in both cases, and as time progresses, our image analysis tool enables us to quantify distinctions between the two hypotheses.

**FIG. 8.**
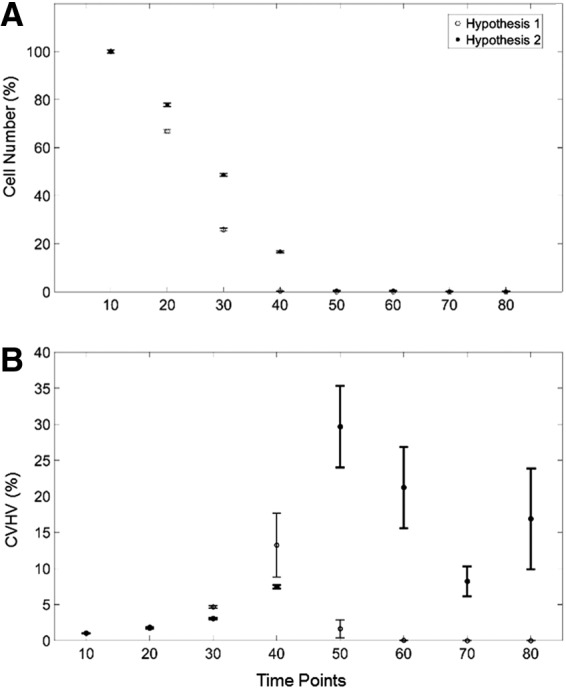
Computational model results (mean ± SE) for **(A)** number of cells, and **(B)** CVHV for cells following the rules for Hypothesis 1 (open circles) and Hypothesis 2 (closed circles) for all 30 images of 2D slices gathered from the 3D model at time points 10, 20, 30, 40, 50, 60, 70, and 80. 2D, two-dimensional.

While we observed a particularly rapid drop-off for both models of around 20 time points, we also observed a faster drop-off of cell numbers in the model for Hypothesis 1 (increased proliferation on higher agent), as seen in Figure 8A, which shows the mean ± SE cell number at each time point determined from 30 independent runs of the model, with the results from each run normalized to its respective Day 1 value. The cell numbers were statistically significantly different for Hypothesis 1 versus Hypothesis 2 (*p* < 0.001).

Figure 8B shows the corresponding trends in CVHV at each time point. Under Hypothesis 1 (shorter lived cells with increased proliferation on higher local substrate concentration), this value initially rises until 40 time points and then trends continually downward. Under Hypothesis 2 (longer lived cells with increased survival on higher local substrate concentration), this value rises until 50 time points, and its values remain high relative to the initial CVHV values. The CVHV values under Hypothesis 1 versus Hypothesis 2 were statistically significantly different (*p* < 0.001). For both figures, as with the experimental cell numbers and corrected Voronoi centroids, we have normalized the values by the value observed at the first time point to focus in the trends.

Cell numbers and CVHV are expressed as percentages of their respective values at the initial time point. At time point 10 under Hypothesis 1, there were 568.90 ± 4.24 engrafted cells with a CVHV value of 3.62 ± 14 × 10^3^, while for Hypothesis 2, there were 596.73 ± 4.76 engrafted cells with a CVHV value of 3.03 ± 0.09 × 10^3^. The sudden apparent increase in SE at time point 40 is due to the very low cell numbers at this point and beyond.

**FIG. 9.**
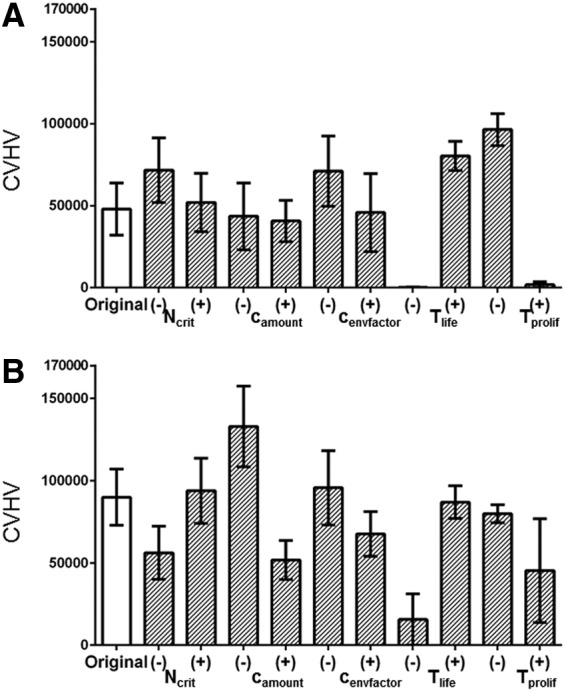
Sensitivity analysis of the model parameters expressed in terms of CVHV following **(A)** Hypothesis 1 measured at time point 40, and **(B)** Hypothesis 2 measured at time point 50.

We show a sensitivity analysis in Figure 9 for the key parameters of the model under each of the two hypotheses, determined by increasing or decreasing each parameter, in turn, by 5% at the beginning of the simulation and then measuring the CVHV (see figure legend for details). We measured these values at time point 40 for Hypothesis 1 and time point 50 for Hypothesis 2, as these are time points at which we judged a dramatic drop in the number of engrafted cells in the original model, as these are time points by which the initial cells must have engrafted and then proliferated or else have died, and thus variation to the model could theoretically have significant effects on its behavior.

One-way ANOVA indicates that altering *T_life_* and *T_prol_* results in statistically significant changes to CVHV for the model under both Hypotheses, which is perhaps not surprising given that these are the two key parameters differentiating cellular behavior between the two Hypotheses. Altering the other parameters resulted in statistically insignificant changes to the CVHV value for either of the two Hypotheses.

The original CVHV value is shown at the far left of Figure 9. The hatched bars show CVHV after increasing (+) and decreasing (−) the attachment probability (*N_crit_*), the amount of collagen each cell adds to the environment (*c_amount_*), and the factor by which the cell lifetimes or proliferation times are influenced by the environment (*c_envfactor_*), the lifetime (*T_life_*), and the proliferation time (*T_prol_*), respectively, by 5%. NS indicates no significant change from control. Asterisk (*) indicates statistically significant change from control.

## Discussion

Stem cell research in the lung has gained considerable momentum recently with the advent of procedures for recellularizing decellularized lung scaffolds. Nevertheless, this research remains largely empirical with few predictive tools available to guide experimentation. As a start to filling this void, we have developed a computational model for testing hypotheses about the rules that might govern cellular engraftment and proliferation on decellularized lung scaffolds. However, given the number and complexity of the cellular decisions that can potentially be made during this process, there is the potential for the number of plausible hypotheses to become so numerous that none of the hypotheses can be supported by model predictions to the exclusion of all the others.

Accordingly, in this study, we apply the model to a very simple experimental scenario about which the rules of cell behavior appear to be limited to local environmental factors, such as geometry and chemistry, determining the probabilities of engraftment, movement, and death. Of course, this model is far from a completely accurate representation of the underlying physiology due to our current lack of knowledge about all the biological details, about which we are forced to make numerous assumptions. Once these assumptions are made, we can calibrate the time scale of the model to actual time (e.g., the time scales of Fig. 8 relative to Fig. 6). Our intent in this study is merely to capture what we believe to be the key aspects of real biological behavior to create a model that has a useful predictive value in terms of the overall behavior of stem cell engraftment on lung scaffolds.

Both C10 cells and MSCs experience a progressive decline in cell numbers over the time course of the experiment (Fig. 7A); so this observation alone does not suggest anything to distinguish the rules governing the behaviors of these two cells. Indeed, the almost complete disappearance of both cell types by the end of the 28th day suggests that the slice culture technique utilized in this study may be insufficient to support appropriate long-term recellularization, highlighting the need for continued investigation of alternative recellularization protocols and procedures. On the other hand, visual inspection of the way in which experimental cell distributions change suggested that there may be differences in the rules of behavior between the two cell types. These distributions suggest that the periphery of a slice of scaffold provides a different cellular environment compared to the scaffold center. We encode this environmental factor in the model by imposing a gradient in the concentration of a generic substrate that the cells are sensitive to, and thus that has the potential to cause the cells to make decisions that are influenced by their locations on the scaffold.

We codified differing tendencies in Hypothesis 1 versus Hypothesis 2, and then determined if the computational model would lead to qualitatively similar predictions based on the rules embodied in these hypotheses. The most straightforward manner in which to compare experiment to prediction is in terms of relative cell numbers. Although having the same overall trends, the MSCs nevertheless decrease in number somewhat more precipitously than the C10 cells (Fig. 7A). By including a greater tendency of cells to survive on regions of higher substrate concentration (Hypothesis 1), the model predicted similar differences in cell numbers (Fig. 9A). These differences are difficult to appreciate simply from visual inspection of the model as time evolves (Fig. 8), demonstrating that quantitative metrics are necessary to objectively test these hypotheses.

Cell numbers alone, however, give no information about the topographical details of the cell populations on the scaffold, yet these details appear to be critical to what distinguishes the two cell types. Accordingly, we devised an algorithm for calculating metrics related to cell topography. We wanted this algorithm to be robust, yet at the same time sensitive to the way in which the cells might be clumped together in groups versus being spread randomly over the scaffold. We felt that these requirements were best met by an algorithm that incorporates a spatially integrated measure of the local neighborhood surrounding each cell. Accordingly, we determined the centroid of the histogram of cell neighborhood areas, the CVHV (Figs. 7B and 9B), because this uses all the information in the histogram to provide a quantity that is robust to noise.

The cell neighborhoods themselves were determined using Voronoi tessellation which produces a plan-filling set of tiles having centroid positions defined by each of the identified cells in the image, and thus provides an intuitively satisfactory way of defining each cell's territory. Nevertheless, the CVHV is still a relatively crude overall reflection of cellular spatial distribution. There are others we could potentially consider that contain more information such as mean distance from some landmark, spatial frequency, or the distribution of distances between pairs of cells. Such metrics might be necessary to test hypotheses about cellular behavior that are more detailed than the two we consider in this study.

The CVHV reveals a key distinction between the two experimental cell types. The CVHV values for C10 cells at later time points remain significantly higher relative to the initial CVHV values (Fig. 6B). This same difference in CVHV was recapitulated in the model by Hypothesis 2 versus Hypothesis 1 (Fig. 8B), supporting the notion that MSCs are more likely to proliferate on the regions of higher substrate concentration, which are located toward the periphery of the scaffold as predicted by Hypothesis 1, while the C10 cells are more inclined to survive on regions of higher substrate concentration as predicted by Hypothesis 2.

These conclusions represent only the very beginning of an elucidation of the enormously complex set of rules governing how cells behave in a complex environment such as the decellularized lung scaffold. Nevertheless, they can now be considered notions that have stood up to the first level of scientific scrutiny, which provides direction for further mechanistic investigation. For example, we can now ask what the topographical substrate on the scaffold might be, the mechanisms by which C10 cells and MSCs respond to it, and potentially what might be manipulated on a scaffold to improve the success of recellularization.

Of course, these conclusions and the questions they generate are contingent upon the limitations of our computational model and our experiments. Such limitations are inevitably considerable given the biological complexities of an actual recellularization scenario, the current paucity of our knowledge about underlying mechanisms and biological parameters, and the need to make simplifications and approximations in the interests of computational tractability. For example, a decellularized scaffold is composed of a large number of extracellular and residual intracellular proteins and proteoglycans,^14,20^ rather than a single functional substrate distributed as a linear gradient.

Also, because sustained recellularization was ultimately unsuccessful in the experimental system employed in this study, we consider only the very initial events in the process, namely cellular attachment, movement, and proliferation. Eventually the engrafted cells must differentiate if they are to successfully lead to a regenerated tissue; so our model will eventually have to incorporate these events into its rule set when suitable experimental data become available.

It is also worth noting that there is still some degree of heterogeneity in the scaffold following decellularization. Studying cellular engraftment on homogeneously constructed artificial scaffolds might allow for more precise delineation of the rules of cellular behavior. On the other hand, the behavior itself might be fundamentally altered by an artificial environment; so determining all the details of the rule sets required for a realistic agent-based model may eventually require a combination of both approaches.

Our computational model thus represents a first step in the objective evaluation of sets of rules of cell governing the dynamic behavior of cells engrafted onto decellularized lung scaffolds. This complements other recent studies on the use of computational modeling to elucidate cellular behavior such as genetic expression in individual stem cells,^21,22^ the role of stem cells in tumor development and metastasis,^23,24^ and embryogenesis in the pancreas.^25^

In conclusion, we have developed an agent-based computational model of cell behavior following engraftment onto decellularized lung scaffold environments. The model suggests that MSCs tend to proliferate on areas of higher local concentrations of substrate when there is an increasing concentration gradient toward the periphery of the scaffold, but without affecting the lifespan. In contrast, the model suggests that C10 cells proliferate uniformly everywhere, but are more likely to survive on areas of higher substrate concentration. It remains to be seen what the actual mechanisms are behind these behavioral rules, but they may suggest where to look.

## Abbreviations Used

ANOVA: analysis of variance
CVHV: Corrected Voronoi Histogram Variance
FBS: fetal bovine serum
MSC: mesenchymal stem cell
P/S: penicillin/streptomycin
SE: standard error
